# Advancing Personalized Interventions: A Paradigm Shift in Psychological and Health-Related Treatment Strategies

**DOI:** 10.3390/jcm13154353

**Published:** 2024-07-25

**Authors:** Luca Cerniglia

**Affiliations:** Faculty of Psychology, International Telematic University Uninettuno, 00186 Rome, Italy; luca.cerniglia@uninettunouniversity.net; Tel.: +39-0669761

## 1. Introduction

In recent years, the field of psychological and health-related interventions has seen a paradigm shift towards personalized and tailored approaches [1,2,3,4]. The papers presented in this Special Issue of the *Journal of Clinical Medicine* reflect the cutting-edge advancements in this domain, emphasizing the importance of individualized treatment strategies to enhance therapeutic outcomes across diverse populations. From culturally sensitive behavioral interventions to gender-specific treatment responses and the integration of genetic and epigenetic insights, these studies collectively underscore the critical need to move beyond one-size-fits-all approaches. This editorial aimed to synthesize the key findings from these studies, highlighting their contributions to the evolving landscape of personalized medicine and psychology.

The study by Zerr, McCabe, Zhang, and Yeh investigates the efficacy of a personalized behavioral parent training (BPT) intervention, specifically parent–child interaction therapy (PCIT), to enhance treatment engagement and outcomes among culturally diverse families. It explores the potential of parent explanatory model (PEM) personalization to address misalignments between parent and treatment expectations, etiological explanations, parenting styles, and family support, which can lead to poor treatment outcomes. This paper highlights that ethnic minorities often face barriers to effective mental health treatment due to misalignment between their beliefs and traditional treatment models. The study introduces the PersIn framework, which customizes PCIT to align with individual PEMs, aiming to improve engagement and outcomes.

In their investigation of gender variations in early weight change and variability throughout inpatient treatment for adolescents with anorexia nervosa (AN), Halbeisen, Braks, Huber, and Paslakis examine this topic. The researchers looked at whether weight change and variability within the first 14 days of therapy varied for young men and women with AN, and if these responses predicted treatment results equally for both genders. According to the study, weight fluctuation and early weight changes are important indicators of how well an AN therapy would go. For both gender groups together, weight changes predicted the discharge weight of patients. Weight fluctuation was associated with increased psychopathology related to disordered eating and anxieties about one’s body upon discharge. Only when it came to weight gain—which was more prominent in young men—did gender differences appear, and gender affected general psychopathology outcomes.

The study by Halidu and Kotera examines the association between adolescent social anxiety, school satisfaction, family emotional support, and school absenteeism. Utilizing data from the Young-HUNT3 study and the Norwegian National Education Database, the researchers aimed to understand how family emotional support can moderate the influence of social anxiety on school satisfaction and absenteeism among adolescents.

The research found that adolescents with higher levels of social anxiety tend to experience lower school satisfaction and higher absenteeism. However, family emotional support significantly moderated these associations. Specifically, family support mitigated the negative effects of social anxiety on school satisfaction and reduced the likelihood of school absenteeism. These findings highlight the critical role of a supportive family environment in alleviating the adverse impacts of social anxiety on adolescents’ school experiences.

The psychological states of obese Italian teenagers seeking an in-hospital multidisciplinary body weight reduction program are examined in the study by Guerrini Usubini, Bottacchi, Bondesan, Frigerio, Marazzi, Castelnuovo, and Sartorio. The objective of the study was to evaluate this population’s psychological adjustment, emotional states, and co-occurring eating disorder symptoms. The results showed that, in comparison to men, women scored higher on the BDI, both STAI subscales, the bulimia subscale of the EAT-26, emotional symptoms, prosocial behaviors, overall problems, and total impact subscales of the SDQ. Furthermore, compared to those without eating disorder symptoms, participants with eating disorder symptoms scored higher on the BDI, the STAI’s two subscales, and the SDQ’s emotional symptoms and overall problems subscale. The COVID-19 pandemic’s effects on women who have dysregulated eating habits and their children’s emotional and behavioral functioning are investigated in Cerniglia and Cimino’s study [5]. In addition to determining whether internalizing/externalizing and dysregulation symptoms in the children increased during the same period, the research sought to determine whether the symptoms of mothers experiencing binge-eating episodes (BEEs) worsened from the pre-pandemic period (T1) to the pandemic period (T2). The results showed that during the pandemic, mothers’ psychopathological symptoms significantly increased. They also showed higher scores for obsessive-compulsive behaviors, phobic anxiety, sadness, and interpersonal sensitivity. Moreover, mothers during the pandemic reported greater rates of emotional eating and uncontrollable eating.

The study by Etindele Sosso, Torres Silva, Queiroz Rodrigues, Carvalho, Zoukal, and Zarate investigates the prevalence of sleep disturbances in Latin American populations and their association with socioeconomic status (SES). This systematic review and meta-analysis aims to document the global prevalence of sleep disturbances in these populations and explore how various SES factors influence different types of sleep disturbances.

One of the main conclusions is that those with higher SES slept for shorter periods of time, while people with lower SES slept for shorter periods of time, had less frequent snores, more common EDS, and sleep bruxism. Higher education levels were linked to greater cases of sleep bruxism, whereas lower education levels were linked to insomnia. A lower prevalence of sleep disturbances was linked to greater education, whereas a higher prevalence was linked to lower income, unemployment, and being a housewife. The combined prevalence of sleep disturbances was 24.73%. This study emphasizes the need for a multifaceted strategy to manage sleep disturbances and recommends that public health initiatives focus on the socioeconomic variables that contribute to differences in sleep quality.

The study by Cunitz, Holloway, Harzendorf, Greving, Zeldovich, Krenz, Timmermann, Koerte, Bonfert, Berweck, Kieslich, Brockmann, Roediger, Buchheim, Andelic, Lendt, Staebler, Muehlan, and von Steinbuechel examines health-related quality of life (HRQoL) after pediatric traumatic brain injury (pTBI) from both children’s and parents’ perspectives using the QOLIBRI-KID/ADO questionnaire. This study aims to assess the degree of agreement between the self-reported HRQoL of children and adolescents and the parental proxy-patient perspective, considering the impact of age, TBI severity, sex, recovery, and the presence of chronic or mental health diseases on this agreement. This study highlights that parental reports should not be solely relied upon to assess HRQoL in children and adolescents after TBI, emphasizing the importance of incorporating children’s self-reports when feasible. This approach is crucial, as parental perspectives often underestimate or overestimate the children’s actual HRQoL, which can influence the overall understanding and subsequent interventions for improving pediatric health care after TBI. The findings advocate for a more integrated use of both child/adolescent and parental perspectives to achieve a comprehensive assessment of HRQoL post TBI.

In their research, Lee, Kim, Ha, and Kim examine how stress recognition mediates the association between generalized anxiety disorder (GAD) and smartphone dependence (SPD) in Korean teenagers. The 16th Adolescence Health Behavior Survey’s (2020) data were used by the researchers to examine 54,948 middle- and high-school students’ answers. According to the degree of their smartphone addiction, the study divided teenagers into three groups: normal, self-control failure (SCF), and serious consequences (SC). The body mass index (BMI), subjective health recognition, happiness, and attempts to regulate weight were all taken into consideration while assessing anxiety levels using the GAD-7 scale. According to the findings, teenagers with greater levels of SPD performed worse academically, were less happy, and perceived stress more. GAD and SPD were positively correlated, with more SPD being associated with more severe GAD symptoms. Moreover, a larger chance of habitual drug use was linked to higher levels of loneliness, depression, suicidal thoughts, intentions, and attempts, as well as heightened feelings of melancholy and loneliness. Gender differences showed that while men were more likely to take drugs, women were more likely to experience depression, despair, and suicide ideation. This study discovered that the relationship between GAD and SPD was mediated by stress detection.

## 2. Conclusions

The collective insights from the studies in this Special Issue highlight a pivotal transformation in our approach to psychological and health-related interventions. The emphasis on personalized treatment strategies, whether through cultural adaptation, gender-specific responses, family dynamics, or genetic and epigenetic profiling, marks a significant advancement in optimizing therapeutic outcomes [6,7,8,9]. These studies underscore the profound impact of tailored interventions in addressing the unique needs of individuals, thereby enhancing their mental and physical well-being.

As we move forward, it is imperative to continue exploring and refining these personalized approaches. Future research should focus on expanding our understanding of the intricate interactions between genetic predispositions, environmental influences, and individual psychological responses. Moreover, interdisciplinary collaboration will be crucial in developing comprehensive intervention models that integrate these diverse factors.

The importance of these topics cannot be overstated, as they hold the potential to revolutionize our approach to mental health and medical treatment [10,11,12,13]. By prioritizing individualized care and leveraging the latest scientific advancements, we can pave the way for more effective, inclusive, and responsive health care systems. This Special Issue serves as a testament to the progress made thus far and a call to action for ongoing innovation and research in the field of personalized interventions [14].
